# A Hybrid Wavelet-Based Method for the Peak Detection of Photoplethysmography Signals

**DOI:** 10.1155/2017/9468503

**Published:** 2017-11-07

**Authors:** Suyi Li, Shanqing Jiang, Shan Jiang, Jiang Wu, Wenji Xiong, Shu Diao

**Affiliations:** ^1^College of Instrumentation and Electrical Engineering, Jilin University, Changchun 130026, China; ^2^The First Hospital of Jilin University, Changchun 130021, China

## Abstract

The noninvasive peripheral oxygen saturation (SpO_2_) and the pulse rate can be extracted from photoplethysmography (PPG) signals. However, the accuracy of the extraction is directly affected by the quality of the signal obtained and the peak of the signal identified; therefore, a hybrid wavelet-based method is proposed in this study. Firstly, we suppressed the partial motion artifacts and corrected the baseline drift by using a wavelet method based on the principle of wavelet multiresolution. And then, we designed a quadratic spline wavelet modulus maximum algorithm to identify the PPG peaks automatically. To evaluate this hybrid method, a reflective pulse oximeter was used to acquire ten subjects' PPG signals under sitting, raising hand, and gently walking postures, and the peak recognition results on the raw signal and on the corrected signal were compared, respectively. The results showed that the hybrid method not only corrected the morphologies of the signal well but also optimized the peaks identification quality, subsequently elevating the measurement accuracy of SpO_2_ and the pulse rate. As a result, our hybrid wavelet-based method profoundly optimized the evaluation of respiratory function and heart rate variability analysis.

## 1. Introduction

Photoplethysmography (PPG) signals are often obtained by using a pulse oximeter. The noninvasive peripheral oxygen saturation (SpO_2_) can be calculated by applying the Lambert-Beer law on the PPG signals and generate the pulse rate simultaneously. SpO_2_ is an important physiological parameter to assess the respiratory function [1, 2]. Previous studies have shown that the pulse rate and pulse-to-pulse interval from PPG are highly correlated with the heart rate and R-R interval from ECG, which indicates that heart rate variability (HRV) analysis can be alternated by pulse rate variability (PRV) analysis [3, 4]. We also did an experiment to obtain the PPG and ECG simultaneously, and the results showed that the alternate is feasible. In other words, the measurement of PPG can obtain not only SpO_2_ and pulse rate, but the PRV analysis, which are the key criteria for the assessment of human respiratory and cardiac autonomic nervous function. Moreover, the pulse sensor is more easily wearable than the ECG sensor.

To date, many experts have made great progress in the domain of PPG denoising and peak detection or heart rate extraction. Since PPG signal is relatively indiscernible and nonstationary, whose collection is inevitable to be mixed with noise and interference, such as high frequency noise, motion artifacts, random noise, and baseline drift [5], high frequency noise and power frequency interference can be effectively suppressed via hardware filter circuit. In this circumstance, the PPG signal denoising mainly focused on the motion artifacts reduction [6–10]. In the field of PPG peak automatic detection, the traditional detection methods were based on derivative approach to locate the local maximum point of the pulse wave [11–13]. Fu et al. [14] indicated that 6-level multiresolution analysis obtained from wavelet transform can effectively extract the heart rate in comparison to that of a moving average approach. Shin et al. [15] developed an improved peak detection algorithm based on adaptive threshold in PPG waveforms. Liu et al. [16] designed a heart rate determination algorithm using the fuzzy logic discriminator to improve the accuracy of the peak detection of the PPG signals. Sun et al. [17] proposed a heartbeat extracting method based on empirical mode decomposition (EMD) and obtained 84.68% detection accuracy of heart rate using PPG signals from PhysioNet database. Kavsaoqlu et al. [18] proposed a peak detection algorithm using adaptive segmentation in raw PPG signals to estimate the heart rate and HRV by comparing with maximum points in these segments.

Although the peaks detection on raw PPG signals will directly reduce the calculation time in the subsequent estimation of SpO_2_ or HRV, the peaks detection includes not only the position but also the amplitude information. Breathing and body movements can cause severe baseline drift and motion artifacts, leading to amplitude changes, resulting in signals with a dispersed, nonstationary, and low-frequency distribution. Therefore, this study focuses on the suppression of the low-frequency noise causing the amplitude changes and on the improvement of the accuracy of the peak identification.

Based on wavelet multiresolution analysis (MRA) principle [19], the signal can be decomposed into a series of details and approximations. The energies of baseline drift and of the partial motion artifacts are mainly concentrated in the approximation component corresponding to the high-level wavelet decomposition of the PPG signal. Therefore, it is feasible to estimate it according to MRA and to get the amplitude more precisely.

The peak identification of PPG can be regarded as the singularity detection problem. Due to the fact that the singularity detection and the quadratic spline wavelet modulus maximum are of great relevance [20], the peak can be identified by using modulus maximum at the decomposition level corresponding to the energy of primary peak wave concentrated.

This study will first introduce the pulse oximeter operation, followed by the subjects' raw signals collection, then describe the principle of the hybrid wavelet-based method and its implementation steps, finally illustrate the evaluation experiment, compare the peak recognition results on the raw signal and on the corrected signal through the ten subjects of PPG signals acquired under sitting, raising hand, and gently walking postures, respectively, and discuss the effectiveness of the proposed method according to the experimental results.

## 2. Materials and Methods

### 2.1. The SpO_2_ Calculation

The calculation of SpO_2_ is derived from Lambert-Beer's law, as shown in

(1) SpO2=A·lg1−ΔImax′/Imax′lg1−ΔImax/Imax+B≈A·ΔImax′/Imax′ΔImax/Imax+B,

where *A*, *B* are certain coefficients which can be determined by calibration experiments; Δ*I*_max_ and Δ*I*_max_′ correspond to the difference between the pulse wave peak and trough under the two kinds of wavelengths of light (generally used 660 nm and 940 nm wavelengths), respectively; *I*_max_ and *I*_max_′ correspond to the pulse wave peak under the above wavelengths of light, respectively. Thus, the peak identification definitely associates with the SpO_2_ calculation, and the accurate positioning of the peak will improve the accuracy of the SpO_2_ calculation.

### 2.2. Device and PPG Signal Acquisition

**Figure 1:**
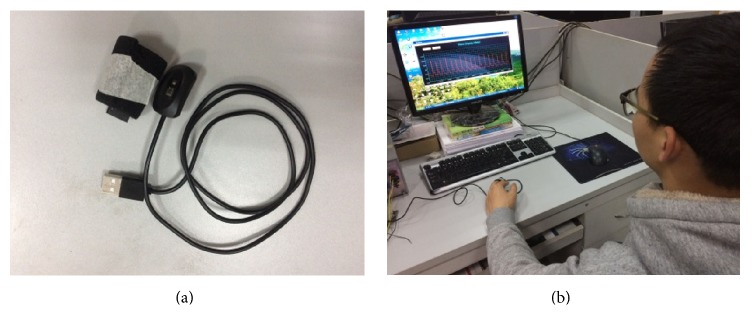
The monitoring scene using the reflective pulse oximeter. (a) The reflective pulse oximeter; (b) the PPG signals collection using the reflective pulse oximeter.

The pulse oximeter used to collect the PPG signals was developed by Tianjin Jingfan Technology Co., Ltd. (see Figure 1(a)). The oximeter is designed based on the reflective photoelectric sensor.  Figure 1(b) shows the monitoring scene using the device.

In this study, 10 healthy volunteers (5 males and 5 females) participated in the experiment, shown in Table 1. The mean age (mean ± std) was 28.7 ± 7.17, the mean body mass index (BMI ± std) was 21.43 ± 3.1. The volunteers were informed about the study before the data was obtained.

**Figure 2:**
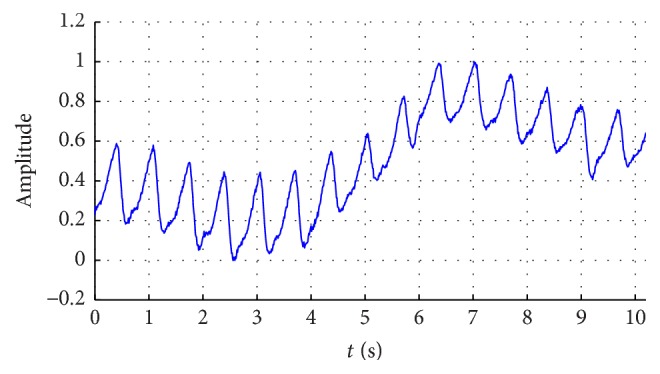
The raw signal of PPG obtained by the reflective probe.

When the subject puts his/her fingertip on the sensor of the device, the raw PPG signals are obtained and recorded.  Figure 2 shows a fraction of the obtained data which has 1024 samples, approximately in 10 s. The amplitude is normalized in the range of 0 and 1. It can be seen from the figure that the high frequency noise in the signal is well suppressed by the hardware filtering, but the signal still has noise due to respiration and movement, affecting the morphologies of the signal.

The quality of PPG signal is relatively good when obtained at rest; however, in dynamic state, the quality will influenced by random noise, motion artifacts, and baseline drift, causing the peak positioning error. Therefore, we will collect volunteers' raw signals in sitting, raising hand, and gently walking postures to test the hybrid peak detection method.

### 2.3. The Hybrid Wavelet-Based Method

The hybrid wavelet-based method mainly includes the suppression and the peaks identification.

#### 2.3.1. The Suppression Method

The suppression aims to lower down the low-frequency noise of PPG signals and to improve the amplitude changing problem caused by baseline drift and partial motion artifacts.

In general, the energy of pulse wave is concentrated in 1–10 Hz. The feature of baseline drift and partial motion artifacts is a kind of nonstationary low-frequency noise, with its energy mainly concentrating in the frequency range less than 1 Hz, which can be approached by applying wavelet MRA theory. Consequently, we can successively decompose the PPG signal to approach the low-frequency noise using the approximation component at the high decomposition level. The method comprises the following steps.

(*1) The Mother Wavelet Selection*. Symlets [21] are compactly supported orthogonal wavelets with the least asymmetry and the highest number of vanishing moments for a given supporting width, and the waveform of sym8's scale function is close to that of the PPG signal; thus the sym8 was chosen as the mother wavelet.

(*2) The Decomposition Level Determination*. The determination of the decomposition level is related to the mother wavelet, sampling rate, and the length of the signal. Since the baseline drift and partial motion artifacts are classed as the nonstationary low-frequency noise, the maximum decomposition level *L*_max_ is regarded as the optimal decomposition level.

(2) $$\begin{array}{c} L_{max}=fix\left[\;\frac{\left(\log\left(N/\left(lw-1\right)\right)\right)}{\log2}\;\right], \end{array}$$

where *N* is the samples of the signal and lw is the length of the wavelet filter. In this application, the sampling rate was 100 Hz and the processing data had 1024 samples each time; the data could be decomposed using sym8 wavelet with 6 decomposition levels.

(*3) The Noise Estimation*. The energy of baseline drift and partial motion artifacts mainly concentrates in the frequency range less than 1 Hz. We can successively decompose the PPG signal to approach the low-frequency noise using the approximation component at level 6 whose frequency range is approximately 0–0.9 Hz.

(*4) The Signal Reconstruction*. The low-frequency noise can be estimated by using the approximation at level 6, and the signal can be corrected by removing the estimated noise from the original PPG signal.

#### 2.3.2. The Peaks Identification Algorithm

The spline wavelet has a better detection effect on the sharp variation points [20], and therefore the peak identification algorithm is designed based on the quadratic spline wavelet modulus maximum algorithm. At the 100 Hz sampling rate, by analyzing the power spectra of the decomposed levels of the quadratic spline wavelet, the energy of the pulse wave was found to be mainly concentrated in level 4 and level 5, and thus the PPG signal was decomposed into 5 levels. The specific detection algorithm steps are as follows.

(*1) Wavelet Decomposition*. The decomposition formula of the signal by using the quadratic spline wavelet is shown as

(3) Aifx=∑n∈ZhnAi−1fx−2i−1nDifx=∑n∈ZgnAi−1fx−2i−1n,

where *i* corresponds to the decomposition level, *n* is the length of the signal, *A*_*i*_*f*(*x*) are the approximations, *D*_*i*_*f*(*x*) are the details, *h*(*n*) and *g*(*n*) are the filtering coefficients of quadratic wavelet, and their initial values are ($0.125\sqrt{2}$, $0.375\sqrt{2}$, $0.375\sqrt{2}$, $0.125\sqrt{2}$) and ($\sqrt{2}$, $-\sqrt{2}$), respectively. In this application, *i* = 1, 2,…, 5, the signal was decomposed into 5 levels: *n* = 1, 2,…, 1024, the length of the signal; that is, the data points analyzed each time were 1024.

(*2) The Threshold Setting*. To avoid the signal abnormality, the samples processed each time were divided into *N* segments, and the threshold *ε* was updated according to the value calculated every segment:

(4) ε=0.5∗mean∑Nmax⁡sigj∗t:j+1∗t−1,j=0,1…N−1,

where *N* = int(*L*/*t*), *L* is length of the signal processed in iteration, and *t* is the number of samples to compute the threshold.

In this application, in the signal processed in iteration, *L* had 1024 samples, *t* was selected as 256 samples due to containing at least one heartbeat, and *N* was 4. The maximum values of each segment were calculated, and then the half of the average value was taken as the threshold value *ε*.

(*3) Modulus Maximum Sequences Calculation*. The positive and negative modulus maximum sequences at level 4 and level 5 were extracted, respectively, by using *ε*, and then the modulus maximum sequences which existed on both two levels were retained.

(*4) The Modulus Maximum Pairs Selection*. Artifacts generally produced isolated maximum points rather than positive and negative maximum pairs, thus removing isolated maximum points from the modulus maximum sequences. In addition, Two pairs of maximum points during 200 ms appearing is not feasible, thereby retaining the pair with largest amplitude. Given all these, the modulus maximum pairs were selected from the modulus maximum sequences.

(*5) The Peak Identification*. The zero-crossing position of the pair relates to the peak position, so we can identify the peak by searching the maximum value around the zero-crossing position in the original signal and the maximum value corresponds to the peak.

## 3. Results and Discussions

In this study, ten subjects' data were collected by the reflective pulse oximeter to evaluate the hybrid method.

### 3.1. The Results of Suppression

**Figure 3:**
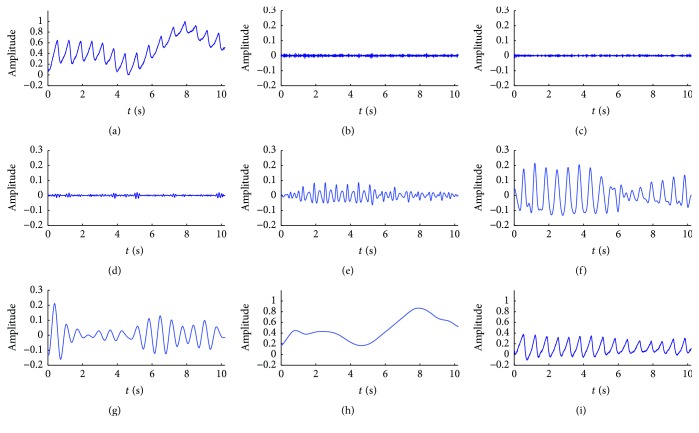
Wavelet decomposition process of PPG signal by using sym8 wavelet at 6 levels. (a) 10-second measured signal of PPG (ppg10s), (b) level 1 details, (c) level 2 details, (d) level 3 details, (e) level 4 details, (f) level 5 details, (g) level 6 details, (h) level 6 approximation, and (i) the corrected signal.

Sym8 wavelet with 6 decomposition levels was applied for the low-frequency noise suppression in PPG signals. To illustrate the method in detail and to see the waveform clarity, a fraction of measured data randomly is set up as* ppg10s*, shown in Figure 3(a). The decomposition process had 6 iterations. After the first decomposition,* ppg10s* was separated into detail at level 1 and approximation at level 1, with successive approximations being decomposed subsequently, so that* ppg10s* was separated into level 1 to level 6 details (see Figures 3(b)–3(g)) and level 6 approximation (see Figure 3(h)). Comparing Figures 3(a) and 3(i), we can see that the amplitude affecting the low-frequency noise is well corrected using level 6 approximation.

### 3.2. Peaks Identification

**Figure 4:**
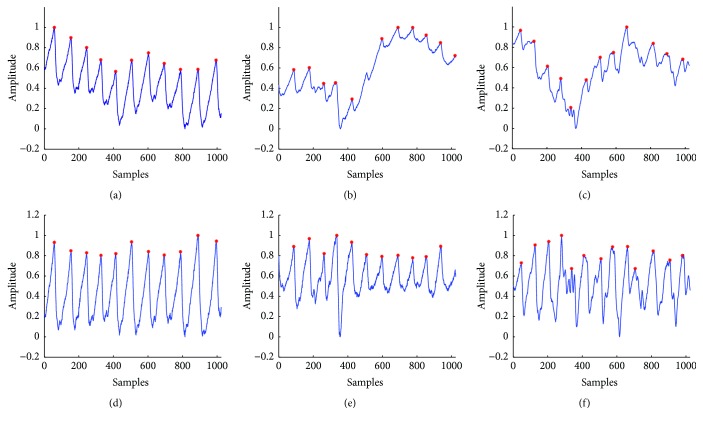
The peak identification results of (a) the raw signal collected in sitting posture, (b) the raw signal collected in raising hands posture, (c) the raw signal collected in gently walking posture, (d) the corrected signal of sitting posture, (e) the corrected signal of raising hands posture, and (f) the corrected signal of gently walking posture.

The peaks were identified on the raw and the corrected PPG signals using the method described in Section 2.3.2, respectively. We recorded the real beats whilst we were doing each experiment, and the reference peaks are positioned by the expert from the First Hospital of Jilin University according to the clinical experience. Figures 4(a), 4(b), and 4(c) were selected volunteers' raw signals collected in sitting, raising hand, and gently walking postures, respectively. Figures 4(d), 4(e), and 4(f) were the corresponding corrected signals in the same postures, respectively. Red stars marked the recognition results of peaks in Figure 4.

However, in raising hand and gently walking postures, the raw signals were seriously affected by baseline drift and motion artifacts, and the recognition accuracy would be affected accordingly. By comparing the peak recognition before correction (see Figures 4(b) and 4(c)) and after correction (see Figures 4(e) and 4(f)), the results indicated that the suppression method played a vital role in improving the recognition accuracy.

Each experiment on the (10) subjects was repeated for 6 times, and each time collected approximately 10 s data of the above three postures. For example, selecting the gently walking posture, Table 2 showed the total peaks detection error (see (5)) of subject (1) on the raw data and on the corrected data, respectively:

(5) $$\begin{array}{c} Error=\frac{FP+FN}{RB}\times 100\%, \end{array}$$

where RB is the real beats recorded, FP is the false numbers detected, and FN is the lost numbers.

**Figure 5:**
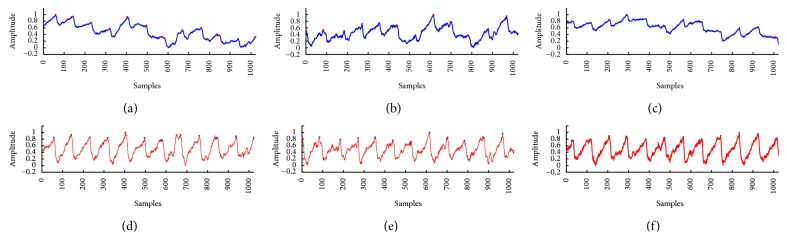
The detection error produced of subject (1) whilst under gently walking posture. (a) The FP produced using the raw data for the 2nd time, (b) the FN produced using the raw data for the 3rd time, (c) the FN produced using the raw data for the 4th time, (d) the FP produced using the corrected data for the 2nd time, (e) no error using the corrected data for the 3rd time, and (f) no error using the corrected data for the 4th time.

In Table 2, the detection algorithm on the raw signal produced a total of 3 errors (4.29%) and that on the corrected signal produced a total of 1 error (1.43%).  Figure 5 illustrated the error locations detected by the algorithm.

Table 3 listed the comparison of the total peaks identification results under the three postures. We can see in sitting posture (total real beats were 761) that the peaks identification results were good whether on the raw signal (100.0%) or on the corrected signal (100.0%); however in raising hand (total real beats were 801) and gently walking (total real beats were 858) postures, the peak recognition accuracies after correction (99.50% and 98.60%) outperformed compared to that before correction (97.88% and 95. 80%).

**Figure 6:**
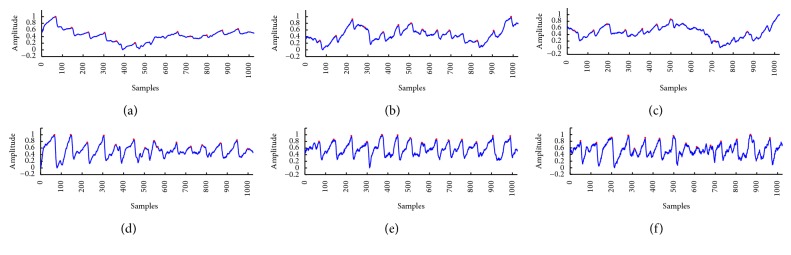
The comparison of the detection results of subject (3) under gently walking posture. (a) The error produced using the raw data for the 1st time, (b) the error produced using the raw data for the 5th time, (c) the FN produced using the raw data for the 6th time, (d) the error produced using the corrected data for the 1st time, (e) no error using the corrected data using the corrected data for the 5th time, and (f) the error produced using the corrected data for the 6th time.

In Table 3, the detection errors of subjects (3), (4), and (10) whilst under gently walking posture were recorded as high; this is mainly due to the big lateral movements whilst walking or heavy breathing during the PPG signal collection. Figures 6(a), 6(b), and 6(c) showed the detection results on the raw PPG signals; we can see the severe distortion in Figure 6(c), causing many detection errors. Figures 6(d), 6(e), and 6(f) were the detection results on the corresponding corrected signals; through the suppression of the low-frequency noise, the morphologies were significantly improved, and hence the detection errors were correspondingly reduced.

## 4. Conclusions

A hybrid wavelet method was proposed to automatically identify PPG peaks. (1) To reduce the influence of low-frequency noise on the signal morphologies, we applied the principle of wavelet multiresolution analysis and determined the mother wavelet and the decomposition level according to the characteristic of the PPG signal and the empirical formula and used the approximate component corresponding to the highest decomposition level to estimate the low-frequency noise of the PPG signal and then obtained the corrected signal. (2) When the PPG signal was decomposed by quadratic spline wavelet, the dominant energy of PPG concentrated in level 4 and level 5 and the high frequency noise mainly in level 1 and level 2. This implied that the modulus maximum pairs generated by peaks can achieve good resolution at the fourth and fifth level; on these grounds, we have designed the identification method which can get better detection accuracy, coupled with the insensitive to high frequency noise. (3) We employed a reflective pulse oximeter developed by Tianjin Jingfan Technology Co., Ltd., to collect the PPG signals under three postures (sitting, raising hand, and walking gently) and compared the peak error detection results on the raw signal (0.0%, 2.12%, and 4.20%) and on the corrected signal (0.0%, 0.50%, and 1.40%), respectively. The results showed that the hybrid method can achieve better identification accuracy and indicated that the method is helpful to improve the accuracy of calculation of SpO_2_ and extraction of PPI subsequently and laid the foundation for the subsequent evaluation of human respiration and the analysis of HRV based on PPG signal.

## Figures and Tables

**Table 1:** The basic personal information of the subjects participated in the experiment.

Subject	Gender	Age (year)	Height (cm)	Weight (Kg)	BMI
(1)	Female	45	161	58	22.4
(2)	Female	24	158	41	16.4
(3)	Male	25	162	52	19.8
(4)	Male	26	162	70	26.7
(5)	Female	26	170	60	20.8
(6)	Male	38	170	62	21.5
(7)	Female	24	162	55	21.0
(8)	Female	24	160	45	17.6
(9)	Male	30	178	78	24.6
(10)	Male	25	169	67	23.5

**Table 2:** An example of the total peaks detection error computed.

Iteration	RB	Raw signal			Corrected signal		
		FP	FN	Error (%)	FP	FN	Error (%)
(1)	12	0	0	0.00	0	0	0.00
(2)	11	1	0	9.09	1	0	9.09
(3)	12	0	1	8.33	0	0	0.00
(4)	12	0	1	8.33	0	0	0.00
(5)	11	0	0	0.00	0	0	0.00
(6)	12	0	0	0.00	0	0	0.00

Total	70	1	2	4.29	1	0	1.43

**Table 3:** The total peaks identification results.

Subject	Sitting			Raising hand			Gently walking		
	RB	Err_R (%)	Err_C (%)	RB	Err_R (%)	Err_C (%)	RB	Err_R (%)	Err_C (%)
(1)	68	0.00	0.00	69	0.00	0.00	70	4.29	1.43
(2)	62	0.00	0.00	70	1.43	0.00	90	0.00	0.00
(3)	74	0.00	0.00	79	5.06	1.27	83	10.84	4.82
(4)	78	0.00	0.00	80	1.25	0.00	83	10.84	2.41
(5)	74	0.00	0.00	77	0.00	0.00	94	1.06	0.00
(6)	70	0.00	0.00	75	1.33	0.00	79	0.00	0.00
(7)	88	0.00	0.00	85	4.71	1.18	89	3.37	1.12
(8)	103	0.00	0.00	104	1.92	0.96	115	0.00	0.00
(9)	78	0.00	0.00	92	3.26	1.09	78	3.85	1.28
(10)	66	0.00	0.00	70	1.43	0.00	77	10.39	3.90

Total	761	0.00	0.00	801	2.12	0.50	858	4.20	1.40

*Note*. Err_R represents the detection error on the raw signals; Err_C means the detection error on the corrected signals.
